# The Effectiveness of Modified Cottle Maneuver in Predicting Outcomes in Functional Rhinoplasty

**DOI:** 10.1155/2014/618313

**Published:** 2014-08-25

**Authors:** Elaine Fung, Paul Hong, Corey Moore, S. Mark Taylor

**Affiliations:** ^1^Department of Surgery, Dalhousie University, 5850 University Avenue, Halifax, NS, Canada B3H 2Y9; ^2^Department of Surgery, IWK Health Centre, 5850/5920 University Avenue, P.O. Box 9700, Halifax, NS, Canada B3K 6R8; ^3^Department of Otolaryngology-Head and Neck Surgery, University of Western Ontario, London, ON, Canada N6B 2P2

## Abstract

*Objective*. To assess the outcomes of functional rhinoplasty for nasal valve incompetence and to evaluate an in-office test used to select appropriate surgical techniques.* Methods*. Patients with nasal obstruction due to nasal valve incompetence were enrolled. The modified Cottle maneuver was used to assess the internal and external nasal valves to help select the appropriate surgical method. The rhinoplasty outcomes evaluation (ROE) form and a 10-point visual analog scale (VAS) of nasal breathing were used to compare preoperative and postoperative symptoms.* Results*. Forty-nine patients underwent functional rhinoplasty evaluation. Of those, 35 isolated batten or spreader grafts were inserted without additional procedures. Overall mean ROE score increased significantly (*P* < 0.0001) from 41.9 ± 2.4 to 81.7 ± 2.5 after surgery. Subjective improvement in nasal breathing was also observed with the VAS (mean improvement of 4.5 (95% CI 3.8–5.2) from baseline (*P* = 0.000)). Spearman rank correlation between predicted outcomes using the modified Cottle maneuver and postoperative outcomes was strong for the internal nasal valve (Rho = 0.80; *P* = 0.0029) and moderate for the external nasal valve (Rho = 0.50; *P* = 0.013).* Conclusion.* Functional rhinoplasty improved subjective nasal airflow in our population. The modified Cottle maneuver was effective in predicting positive surgical outcomes.

## 1. Introduction

Chronic nasal obstruction can be quite distressing and often has a negative impact on quality of life [1]. Commonly recognized anatomic factors contributing to nasal obstruction include external nasal deformity, septal deviation, turbinate hypertrophy, and nasal tip ptosis [1]. Further, it is crucial that the anatomic areas involving the external and internal nasal valves are evaluated diligently in the preoperative setting to identify the specific area of obstruction.

The nasal valves have been proposed to be a major regulator of nasal airflow, causing resistance and preventing airflow from exceeding the capacity to warm and humidify inspired air [2, 3]. The external nasal valve describes an area of the nasal vestibule bounded by the alar rim, nasal sill, and caudal septum [4, 5]. The internal nasal valve is bounded medially by the nasal septum, superiorly and laterally by the caudal margin of the upper lateral cartilage, and more laterally by the anterior portion of the inferior nasal turbinate [4, 6]. Many underlying static and dynamic factors can contribute to the obstruction of the nasal valves, including trauma, previous surgery or radiation, congenital weakness of the nasal cartilage, or aging [1, 4]. The Bernoulli principle states that air flowing through a narrow segment accelerates, leading to a decrease in intraluminal pressure [4]. This phenomenon is demonstrated by the collapse of the lateral nasal wall, particularly during deep inspiration. Thus obstruction of the nasal valves may result from varying degrees of static obstruction and dynamic collapse.

Functional rhinoplasty is a well-accepted surgical intervention for correction of nasal airflow obstruction [1–7]. Some commonly used procedures include batten grafts for external valve collapse and spreader grafts for internal valve compromise; other techniques include butterfly onlay grafts, alar rim grafts, suture suspension, and flaring sutures [2–10]. Accurate preoperative diagnosis of the specific anatomic problem and subsequent selection of the appropriate surgical technique will ensure the best possible results for patients undergoing functional rhinoplasty. However, establishing correlation between subjective nasal airflow and physiologic nasal airflow is often difficult [11]. This is certainly observed in many clinic or office settings, where no objective instruments or methods exist to document nasal airflow. Therefore, an easily accessible and reproducible method to examine nasal airflow changes in the clinical setting is needed.

The modified Cottle maneuver [12] may be a simple and easily applied method to determine the presence of external and/or internal nasal valve compromise in an office setting. It may direct surgical planning and lead to improved functional outcomes.

The objectives of the present study are (1) to determine the functional outcomes of rhinoplasty using validated measures and (2) to assess the correlation between the preoperative nasal airflow testing (the modified Cottle maneuver) and postoperative outcomes.

## 2. Methods

A prospective observational study was conducted at the Queen Elizabeth II Health Centre in Halifax, Nova Scotia. Local Institutional Review Board approval was obtained prior to starting this study.

During the study period of 2002 to 2010, patients with longstanding nasal obstruction evaluated by the senior surgeon (SMT), who met the inclusion criteria, were asked to participate. Inclusion criteria included all patients who were selected for surgical correction of an anatomic nasal defect with minimal benefit from medical therapies. Specifically, failed medical therapies included topical nasal corticosteroid sprays and/or nasal saline rinses. All patients were on at least two-month trial of these medical therapies. Regarding surgical correction, patients deemed to require isolated spreader or batten grafts were selected for the study. Patients were excluded if they underwent a combination of surgical techniques, including septoplasty, inferior turbinate reduction, or tip rhinoplasty. As well, patients who used topical nasal medications or had nasal surgery in the past were also excluded. Informed consent was obtained from the participants.

Prior to physical examination, participants were required to complete the rhinoplasty outcomes evaluation (ROE) form and a 10-point visual analog scale (VAS), which rated the subjective nasal airflow of each nostril on a likert scale (as shown below). The ROE is a validated quality of life survey that is useful in evaluating rhinoplasty outcomes [13]. The VAS is also a simple and reliable method to assess nasal obstruction [14].

The rhinoplasty outcomes evaluation form and the 10-point visual analog scale for nasal valve obstruction are shown below. On the VAS, a score of 0 indicates complete obstruction, and a score of 10 indicates complete nasal patency.

1. How well do you like the appearance of your nose?
Not at allSomewhatModeratelyVery muchCompletely01234
2. How well are you able to breathe through your nose?Not at allSomewhatModeratelyVery muchCompletely01234
3. How much do you feel your friends and loved ones like your nose?Not at allSomewhatModeratelyVery muchCompletely01234
4. Do you think your current appearance limits your social or professional activities?Not at allSomewhatModeratelyVery muchCompletely01234
5. How confident are you that your nasal appearance is the best that it can be?Not at allSomewhatModeratelyVery muchCompletely01234
6. Would you like to surgically alter the appearance or function of your nose?Not at allSomewhatModeratelyVery muchCompletely01234
7. How well do you breathe through your nose?No airflowPerfect airflow012345678910
8. Pre-op assessment of baseline, external, and internal nasal valve scores. Baseline Pre-opExternalInternalPost-opLeft    Right    

**Figure 1:**
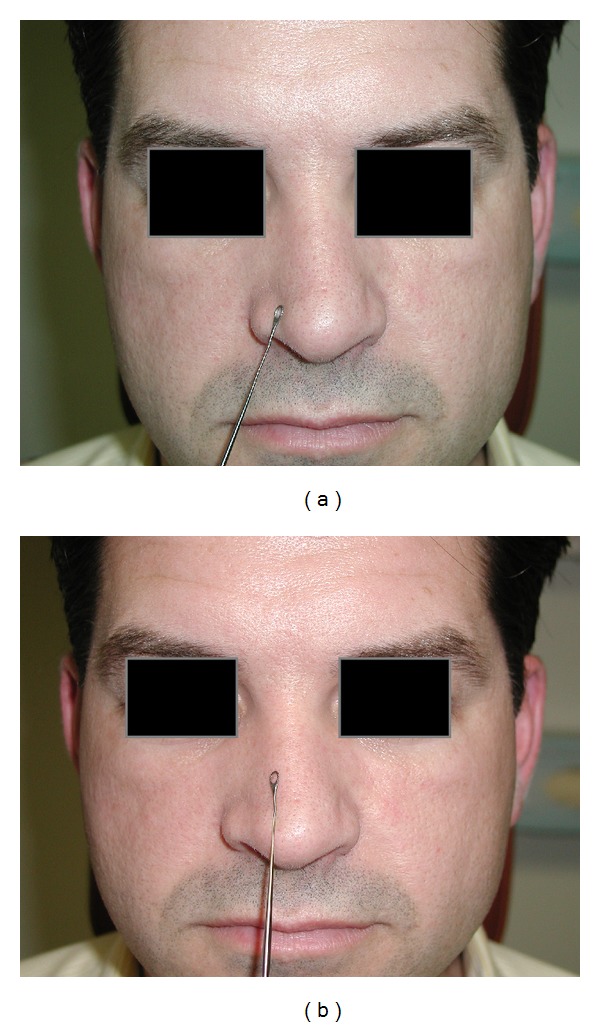
Demonstration of the modified Cottle nasal valve maneuvers ((a) = external valve; (b) = internal valve), with the curette placed exteriorly only to demonstrate the area to be supported intranasally.

The physical examination then commenced, which did not involve the use of topical nasal decongestive medication. Each side of the nose was evaluated independently by occluding the nontest side. All participants were assessed using the modified Cottle maneuver [12]. To test the external valve, an ear curette was used to gently hold the lateral crus of the lower lateral cartilage, and the patient assessed the nasal airflow on the VAS. Another VAS score was obtained for the internal valve when the curette was used to gently lift the upper lateral cartilage of the tested nostril while the patient reassessed the nasal airflow on that side (Figure 1). To improve consistency of this maneuver, only the senior author performed this test with the patients. The modified Cottle maneuver was used to assess nasal valve incompetence and thus select which operative technique was appropriate (batten and/or spreader grafts). Furthermore, the maneuver was used to predict postoperative outcomes.

Spreader grafts were used to correct internal nasal valve collapse; they were all inserted via an open approach. Briefly, the upper lateral cartilages were divided from the septum while preserving the mucoperichondrium to support the spreader graft. A rectangular shaped cartilage graft was then harvested from the nasal septum and placed to span the osteocartilaginous junction to a point caudal to the anterior septal angle. The graft was secured with 5–0 PDS suture (Ethicon, Inc., Somerville, NJ) using a horizontal mattress technique.

Batten grafts were used to strengthen and stabilize the external nasal valves. Briefly, submucosal pockets were dissected in the scroll area just cephalic to the caudal upper lateral cartilage. Cartilage grafts, harvested from the nasal septum, were then placed above the lateral crus and extended laterally toward the piriform aperture. The grafts were secured with 5–0 PDS suture (Ethicon, Inc., Somerville, NJ).

At the 6-month postoperative visit, each patient completed the ROE and VAS as before. The ROE scores were tabulated using the method described by Alsarraf [13]. Both the surgeon and patient were blinded with regard to the preoperative scores. The postoperative nasal patency scores were compared to the preoperative baseline scores, as well as those measuring external and internal valve defects. No surgeon-specific outcome measures were utilized; that is, only the patients assessed the ROE and VAS pre- and postoperatively.

Statistical analysis was carried out using SPSS, version 20 [15]. Overall mean scores were distributed across a wide range and there was statistical evidence that they followed normal distribution, thus allowing the use of the* t*-test to compare the mean scores. Spearman rank correlation was also used in the analysis.

## 3. Results

Forty-nine patients completed the pre- and postoperative ROE questionnaires, along with the in-office assessments using the VAS. There were 24 males and 25 females; mean age was 41 years. There were 35 isolated spreader or batten grafts (24 batten grafts, 11 spreader grafts); the remainder required other adjunctive procedures, such as septoplasty or turbinate reduction. Overall, 92% of the patients reported subjective improvement in their nasal breathing, while 8% did not report any improvement.

The overall ROE score, calculated as (ΣPoints/24 × 100), for each patient was ascertained [13]. The mean ROE score increased from 41.9 to 81.7 (*P* < 0.0001) after surgery.

The mean preoperative and postoperative nasal airflow scores on VAS increased from 3.4 (SD ± 2.3) to 8.0 (SD ± 1.8) after surgery, with a mean improvement of 4.5 (95% CI 3.7–5.3) over baseline. This was statistically significant (*P* = 0.000). The range varied from 1 to 9 points (*P* < 0.0001) on a 10-point scale.

**Figure 2:**
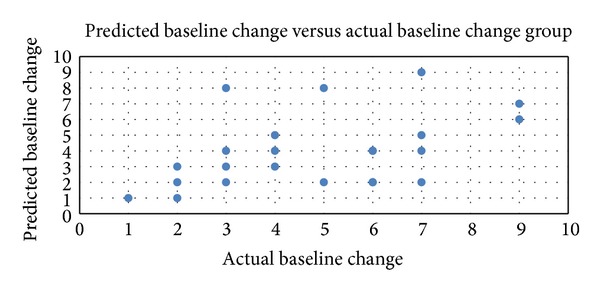
Overall outcomes: Spearman rank correlation scatterplot between predicted outcomes using the modified Cottle maneuver and postoperative outcomes (*N* = 35; Rho correlation = 0.51; *P* = 0.0016, with correlation > 0.05 considered significant).

The predicted mean improvement in nasal airflow using the modified Cottle maneuver for both internal and external valves and the mean improvement in postoperative outcomes were similar (4.1 (SD ± 2.3) versus 4.5 (SD ± 2.2), Table 1). Overall, Spearman rank correlation between the predicted outcomes using the modified Cottle maneuver and the postoperative outcomes showed moderate correlation (Figure 2).

**Figure 3:**
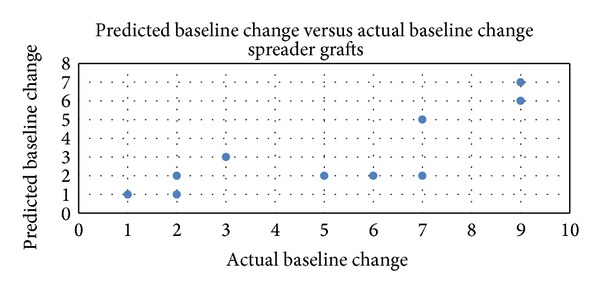
Internal nasal valve: Spearman rank correlation between predicted outcomes using the modified Cottle maneuver and postoperative outcomes using spreader grafts (*N* = 11; Spearman rank correlation = 0.80; *P* = 0.0029, with correlation > 0.05 considered significant).

When the nasal valves were assessed independently, there was a statistically significant correlation between the predicted outcomes and the postoperative outcomes. The predicted mean improvement in internal nasal valve assessment using the modified Cottle maneuver and the actual postoperative outcome were 3.0 (SD ± 1.8) and 5.2 (SD ± 2.8), respectively, (Table 1). The surgical outcome was slightly better than initially predicted (*P* = 0.02). The Spearman rank correlation was found to be strong (Figure 3).

**Figure 4:**
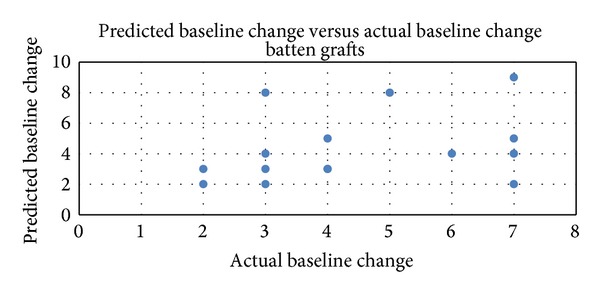
External nasal valve: Spearman rank correlation between predicted outcomes using the modified Cottle maneuver and postoperative outcomes using batten grafts (*N* = 24; Spearman rank correlation = 0.50; *P* = 0.013, with correlation > 0.05 considered significant).

The predicted outcome using external nasal valve maneuver was 3.7 (SD ± 2.3). Similarly, the postoperative outcome was 4.3 (SD ± 1.8) (Table 1). The Spearman rank correlation between the predicted outcome and the postoperative outcome for the external nasal valve was found to be moderate (Rho = 0.50; *P* = 0.013) (Figure 4).

## 4. Discussion

Patients with chronic nasal obstruction often remain a challenge for the surgeon. During the initial preoperative evaluation, it may be difficult to establish correlation between subjective assessment of nasal airflow and actual physiologic and anatomic characteristics of the patient [16–19]. We have examined the effect of a simple office-based test to assess the nasal valves and its relation to postoperative outcomes. Specifically, we used the modified Cottle maneuver to diagnose internal and external nasal valve incompetence in our study population.

The traditional Cottle maneuver is performed by pulling the cheek laterally to assess ipsilateral nasal patency [12]. The modified Cottle maneuver is more precise in the fact that it assesses the upper and lower lateral cartilage support. We used an ear curette to gently lift the upper and lower lateral cartilages individually to specifically identify internal or external nasal valve insufficiency [12]. Our use of the ear curette and the patient-reported VAS score during the preoperative examination of the functional rhinoplasty patient was a simple and reliable tool in predicting postoperative results for those with nasal valve incompetence, as demonstrated by the good correlation values. The advantage of VAS is that it has been shown to correlate with other objective measures of nasal airflow, such as rhinospirometry [20], and each side of the nasal cavity can be tested independently, and, hence, unilateral symptoms can be assessed. Furthermore, each nasal valve can be tested independently [21]. Interestingly, several studies have shown better correlation between VAS than some objective measures for nasal obstruction when unilateral VAS is used [21]. Therefore, the modified Cottle maneuver in our study population was an effective predictor of postoperative outcomes, based on the significant correlation of VAS scores and postoperative results for both spreader and batten grafts.

In addition to the VAS, the ROE scores also demonstrated statistically significant improvements in nasal airflow and quality of life after functional rhinoplasty. The mean ROE score improved from 41.9 to 81.7 (*P* < 0.0001), which is consistent with other published data on primary or revision rhinoplasty procedures [10, 22].

The use of an ear curette to retract the upper lateral cartilage during an evaluation can be uncomfortable for the patient. Hence, introduction of the curette in this sensitive part of the nose may alter the subjective perception of airflow by the patient, and, despite some improvement, it may render the test less reliable in predicting postoperative outcome. However, all patients in our study tolerated this maneuver well and no problems were reported.

A recent systematic review conducted by Rhee and colleagues assessed the evidence supporting functional rhinoplasty and nasal valve repair [10]. Of the 44 articles reviewed, only six (14%) reported outcomes using a validated patient-reported questionnaire and 75% of these studies used adjunctive surgical procedures in combination with nasal valve surgery. Others have noted the difficulty in analyzing outcomes in functional rhinoplasty due to the use of many different surgical techniques, as well as the use of adjunctive procedures, which inevitably leads to confounders [4]. Some surgeons have concluded that patient-oriented measures are generally more effective than objective measures at deducing postoperative outcomes in functional rhinoplasty [4]. Our study is a prospective study using both the ROE and the VAS in isolated batten or spreader graft nasal valve procedures, thus strengthening the evidence for use of these functional rhinoplasty techniques for nasal valve incompetence.

Our study is limited by the nature of the evaluation tool, which is subjective. That is, the modified Cottle maneuver is a subjective test to administer since the degree of elevation or lift of the nasal structures is operator dependent. For instance, significant lifting may always improve nasal airflow, regardless of the site of obstruction. The use of rhinomanometry, acoustic rhinometry, and other tools to objectively assess nasal airflow may be more helpful in the preoperative assessment [20, 21, 23, 24]. However, there are often practical and logistical difficulties associated with their use, especially in a clinic or office setting. They require specialized equipment and an experienced operator; therefore, these tools are not widely used [21]. In addition, these objective measures typically do not specify the location of the nasal obstruction. Hence, a simple assessment tool, even with its subjective imprecision, may be the most practical in the clinical setting. As well, experienced plastic surgeons may develop consistency with the modified Cottle maneuver that may mimic the surgical changes to be imparted by the rhinoplasty procedure.

## 5. Conclusion

The modified Cottle maneuver was used to identify the specific site of nasal valve obstruction. Overall, this assessment was well tolerated and was able to predict positive outcomes in patients who underwent functional rhinoplasty in our patient population. Although modified Cottle maneuver is a subjective test and is operator dependent, it may serve as a simple preoperative assessment tool in patients with nasal valve obstruction.

## Figures and Tables

**Table 1:** Comparison of pre- and postoperative nasal airflow improvement scores on a 10-point visual analog scale for batten and spreader grafts. The preoperative score is obtained while performing the modified Cottle maneuver for both the internal and external nasal valve assessments.

Operative technique	Predicted mean improvement	Mean postoperative improvement	Difference in means	Correlation (>0.05 is significant)
Overall (*N* = 35)	4.1 (SD ± 2.3)	4.5 (SD ± 2.2)	*P* = 0.23	0.51 (*P* = 0.0016)
Internal nasal valve spreader graft (*N* = 11)	3.0 (SD ± 1.8)	5.2 (SD ± 2.8)	*P* = 0.02*	0.80 (*P* = 0.0029)
External nasal valve batten graft (*N* = 24)	3.7 (SD ± 2.3)	4.3 (SD ± 2.0)	*P* = 0.17	0.50 (*P* = 0.013)

∗Refers to statistical significance.
